# Impact of Evolving Treatment Patterns on Interstitial Lung Disease Progression in Systemic Sclerosis Using the European Scleroderma Trials and Research Database

**DOI:** 10.1002/art.70043

**Published:** 2026-03-29

**Authors:** Corrado Campochiaro, Marie‐Elise Truchetet, Madelon Vonk, Giacomo De Luca, Giovanna Cuomo, Lidia P. Ananieva, Eric Hachulla, Vanessa Smith, Ana Maria Gheorghiu, Radim Becvar, Patricia Carreira, Nicolas Hunzelmann, Daniel E. Furst, Vera Ortiz‐Santamaria, Francesco Del Galdo, Marco Matucci‐Cerinic, Anna‐Maria Hoffmann‐Vold, EUSTAR collaborators

**Affiliations:** ^1^ Unit of Immunology, Rheumatology, Allergy and Rare Diseases, Inflammation, Fibrosis and Ageing Initiative, Scientific Institute for Research Hospitalization and Healthcare San Raffaele Hospital Vita‐Salute San Raffaele University Milan Italy; ^2^ Department of Rheumatology University of Bordeaux Bordeaux France; ^3^ Department of Rheumatology Radboud University Medical Center Nijmegen the Netherlands; ^4^ Department of Rheumatology University of Naples Federico II Italy; ^5^ V.A. Nasanova Institute of Rheumatology Moscow Russia; ^6^ Department of Internal Medicine and Clinical Immunology, Referral Centre for Rare Systemic Autoimmune Diseases North of France, North‐West, Mediterranean and Guadeloupe, Centre Hospitalier Universitaire de Lille University of Lille, French National Institute of Health and Medical Research Lille France; ^7^ Department of Rheumatology Ghent University Hospital Ghent Belgium; ^8^ Department of Rheumatology Bucharest University Emergency Hospital Bucharest Romania; ^9^ Department of Internal Medicine Charles University Prague Czech Republic; ^10^ Department of Rheumatology Hospital Universitario 12 de Octubre Madrid Spain; ^11^ Department of Dermatology University Hospital of Cologne Cologne Germany; ^12^ David Geffen School of Medicine University of California Los Angeles; ^13^ Rheumatology Unit Vall d'Hebron University Hospital Barcelona Spain; ^14^ Department of Rheumatology Leeds Teaching Hospitals NHS Trust Leeds United Kingdom; ^15^ Department of Rheumatology Oslo University Hospital Oslo Norway; ^16^ Department of Rheumatology University Hospital Zurich, University of Zurich Zurich Switzerland

## Abstract

**Objective:**

The treatment landscape for systemic sclerosis‐associated interstitial lung disease (SSc‐ILD) has evolved with increasingly available immunosuppressive therapies (ISTs) and antifibrotic treatments. However, their real‐world use remains unclear. The objective of this study was to analyze treatment trends and the effect of IST and antifibrotic treatments on ILD progression using the European Scleroderma Trials and Research database.

**Methods:**

We included patients with SSc‐ILD meeting the 2013 American College of Rheumatology/EULAR criteria with high‐resolution computed tomography–confirmed ILD, pulmonary function, and therapy data, grouped into four time periods (≤2006, 2007–2011, 2012–2016, and ≥2017). We analyzed IST initiation, switching, discontinuation, and combination therapy. ILD progression was defined as a decline in the percentage of predicted forced vital capacity of 5% or greater or the percentage of predicted diffusing capacity of the lungs for carbon monoxide of 10% or greater over 12 ± 3 months.

**Results:**

Among 1,409 patients, IST use at first evaluation increased significantly from 13.6% (≤2006) to 57.4% (≥2017) (*P* < 0.001). Mycophenolate mofetil emerged as the most prescribed IST (7% to 57%) (*P* < 0.001). Combination therapy rose from 17.9% to 26.9% (*P* < 0.001), whereas ILD progression rates declined from 21.3% (2007–2011) to 12.1% (≥2017) (*P* < 0.001). In the 2017 and later cohort, logistic regression showed shorter disease duration (odds ratio [OR] 0.991, 95% confidence interval [CI] 0.987–0.996; *P* < 0.001) and myositis (OR 9.9, 95% CI 1.94–51.76; *P* = 0.006) were associated with therapy initiation, whereas switching was higher in patients with a higher modified Rodnan skin score (OR 1.03, 95% CI 1.00–1.06; *P* = 0.035) and in patients with arthritis (OR 3.03, 95% CI 1.55–5.94; *P* = 0.001). Last, combination therapy was associated with younger age, higher dyspnea class, and arthritis.

**Conclusion:**

Our findings reveal a significant evolution in clinical practice. However, continued disease progression emphasizes the need for more effective therapeutic approaches.

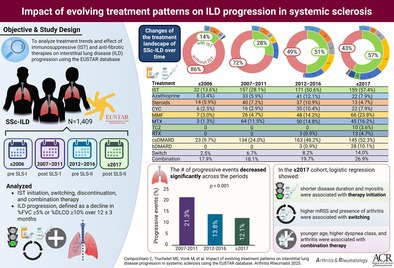

## INTRODUCTION

Interstitial lung disease (ILD) is a major complication in systemic sclerosis (SSc) and contributes significantly to both morbidity and mortality.^1^, ^2^, ^3^ Over the past two decades, the treatment landscape for SSc‐ILD has evolved considerably, with growing recognition of the role of immunosuppression.^4^, ^5^ The pivotal Scleroderma Lung Study (SLS) I in 2006 demonstrated the benefits of cyclophosphamide (CYC) in stabilizing lung function in patients with SSc‐ILD compared with placebo.^6^ This was later confirmed by the SLS II in 2016, which not only reinforced the role of immunosuppression but also established mycophenolate mofetil (MMF) as the cornerstone for SSc‐ILD given its reduced toxicity compared with CYC.^7^ Since then, additional treatment options, including tocilizumab,^8^, ^9^ rituximab (RTX),^8^, ^10^ and nintedanib,^11^ have been investigated. The approvals of tocilizumab by the US Food and Drug Administration in 2021 and nintedanib worldwide in 2019, further impacted the management of SSc‐ILD.^12^ Furthermore, the results of a randomized controlled trial (RCT) comparing RTX with CYC and a RCT performed in Japan comparing RTX with placebo, which led to the approval of RTX in Japan, have solidified the use of RTX in the armamentarium of SSc‐ILD.^8^, ^10^ Based on this evidence, the recently updated EULAR recommendation on SSc management suggested four immunosuppressive drugs (MMF, CYC, RTX, and tocilizumab) and one antifibrotic drug (nintedanib) for the treatment of SSc‐ILD.^13^

Based on the increasing number of available treatments, several key questions evolve, such as the impact of novel therapies on the treatment landscape, particularly regarding the modification of treatment patterns. Given that disease severity, extrapulmonary involvement, and comorbidities play a crucial role in treatment decisions, understanding their impact on treatment choices over time is critical.^14^, ^15^ Moreover, with novel therapies being introduced, it is crucial to determine whether they are truly integrated into clinical practice.^16^ Importantly, the increasing treatment armamentarium has major implications for the design of future SSc‐ILD RCTs.^17^ It is therefore critical to determine the specific treatments currently used and to assess the disease behavior on these treatments. This study aims to provide a comprehensive analysis of the treatment patterns for SSc‐ILD using real‐life data from the European Scleroderma Trials and Research (EUSTAR) database to generate important and novel insights to shape the design of future SSc‐ILD trials, particularly regarding the allowance of background therapy.

## METHODS

### Study design and population

Patients with SSc from the EUSTAR database fulfilling the 2013 American College of Rheumatology/EULAR SSc classification criteria^18^; with radiologically confirmed ILD based on high‐resolution computed tomography (HRCT); available pulmonary function tests, including percentage of predicted forced vital capacity (%pFVC) and percentage of predicted diffusing capacity of the lungs for carbon monoxide (%pDLco); and treatment information at baseline and after 12 ± 3 months were included in the study (EUSTAR CP number 138). The structure of the EUSTAR database and minimum essential data set have been described previously.^19^, ^20^

The study population was segregated into four five‐year long distinct cohorts based on the period in which patients were first evaluated and treated. We segregated the periods based on the first evidence‐based medications assessed in randomized clinical trials for SSc‐ILD, CYC in SLS I, and mycophenolate in SLS 2:

1. ≤2006: pre SLS I study publication.^6^
2. 2007–2011: post SLS I study publication.^6^
3. 2012–2016: pre SLS II study publication.^7^
4. ≥2017: post SLS II study publication.^7^

Each patient was assigned to one of these four periods based on the calendar year of their first EUSTAR assessment (index visit). Patients were then observed longitudinally, but all subsequent visits and outcomes were attributed to that index period.

### Data collection and treatment patterns

Data collected included patient demographics (age, sex, disease duration), clinical characteristics (SSc subtype, SSc autoantibody profile, organ involvement), smoking status, and baseline lung function, including %pFVC and %pDLco. Treatment data included type and duration of immunosuppressive therapies (ISTs) and antifibrotic treatments, and treatment modifications (initiation, switching, and discontinuation).

IST included a daily prednisone dose of at least 10 mg, conventional synthetic disease‐modifying antirheumatic drugs (csDMARDs) (CYC, MMF, methotrexate, and azathioprine), and biologic DMARDs (bDMARDs) (RTX, tocilizumab, and abatacept). Antifibrotic agents included nintedanib and pirfenidone. The following definitions for treatment modifications were used.

- Treatment switching: change from one therapy to another during follow‐up.
- Treatment discontinuation: stopping the primary therapy without initiation of another therapy.
- Unmodified treatment: no change in therapy throughout the study period.
- Combination therapy: the concurrent use of two or more therapies for at least three consecutive months.

### Outcome measures

The primary objective of this study was to assess treatment patterns and patient characteristics of treated patients from before 2006 until today. Treatment patterns included initiation of treatment at patients’ first assessment, switches, discontinuations, and combination therapies compared across the four treatment periods. The secondary objective was to assess the impact of ISTs and antifibrotic treatments over a three‐year follow‐up period on ILD progression. Follow‐up for each patient began at the index EUSTAR and ended after the index visit, death, lung transplantation, or the last EUSTAR visit available within that 36‐month window at the earliest. Visits beyond 36 months were not considered for outcome analyses. ILD progression was defined as a decline in %pFVC of 5% or greater or a decline in %pDLco 10% or greater over 12 ± 3 months. As subanalysis, we assessed ILD progression in antitopoisomerase‐I antibody (ATA) positive patients to enrich for a more severe SSc‐ILD cohort and because these patients are preferably included in RCTs.

### Contemporary cohort combination therapy analysis

To inform the design of future RCTs we assessed current treatment patterns in patients from the latest, contemporary period (≥2017). The analyses included a detailed description of the different treatment courses with a focus on combination therapies and clinical characteristics associated with treatment initiation, switch, discontinuation, and combination. We defined “treatment course” as any course of therapy with a specific drug or combination of drugs per individual patient. To reduce variability, we applied uniform mapping rules (generic names only; combination defined by ≥3‐month overlap) and excluded courses with unresolved inconsistencies.

### Statistical analysis

Descriptive statistics were used to summarize baseline characteristics and treatment patterns across the four periods using means ± standard deviations and proportions, as appropriate. Continuous variables were analyzed using analysis of variance for normally distributed data and Kruskal‐Wallis tests for nonnormally distributed data. Categorical variables were compared using chi‐square or Fisher exact tests, as appropriate. Bonferroni's correction was applied to counteract the problem of multiple comparisons.

Univariable and multivariable logistic regression analyses with odds ratio (OR) and 95% confidence interval (CI) were applied to analyze the impact of clinical characteristics on IST and antifibrotic therapy introduction, switch, discontinuation, and combination therapy in the contemporary period. Given that our main focus was ILD, candidate baseline variables associated with treatment modifications were selected based on reports from the published literature and expert opinion on the factors associated with severe ILD and/or ILD progression: sex, age, reflux/dysphagia symptoms, SSc subtype, antibody status (ATA, anticentromere antibody, anti‐RNA polymerase III antibody [ARA]), baseline %pFVC, baseline %pDLco, disease duration, skin involvement measured by modified Rodnan skin score (mRSS), C‐reactive protein elevation, dyspnea class, synovitis, and myositis. Missing data on the candidate predictors were not imputed. Variables were included if missing values were lower than 30%. In multivariable models, variables were selected with a threshold of *P* = 0.1, and 10 events per variable were needed. All analyses were performed using SPSS Statistics version 26 (IBM, Armonk, NY). Missing values were reported but not imputed. A *P* value of <0.05 was considered statistically significant.

Local ethics committees (when required according to local legislation) of the respective EUSTAR centers approved the collection of data. Data are available on reasonable request. Anonymized data can be shared based on EUSTAR approval and regulations.

## RESULTS

### Changes in immunosuppressive treatments over time

**Figure 1:**
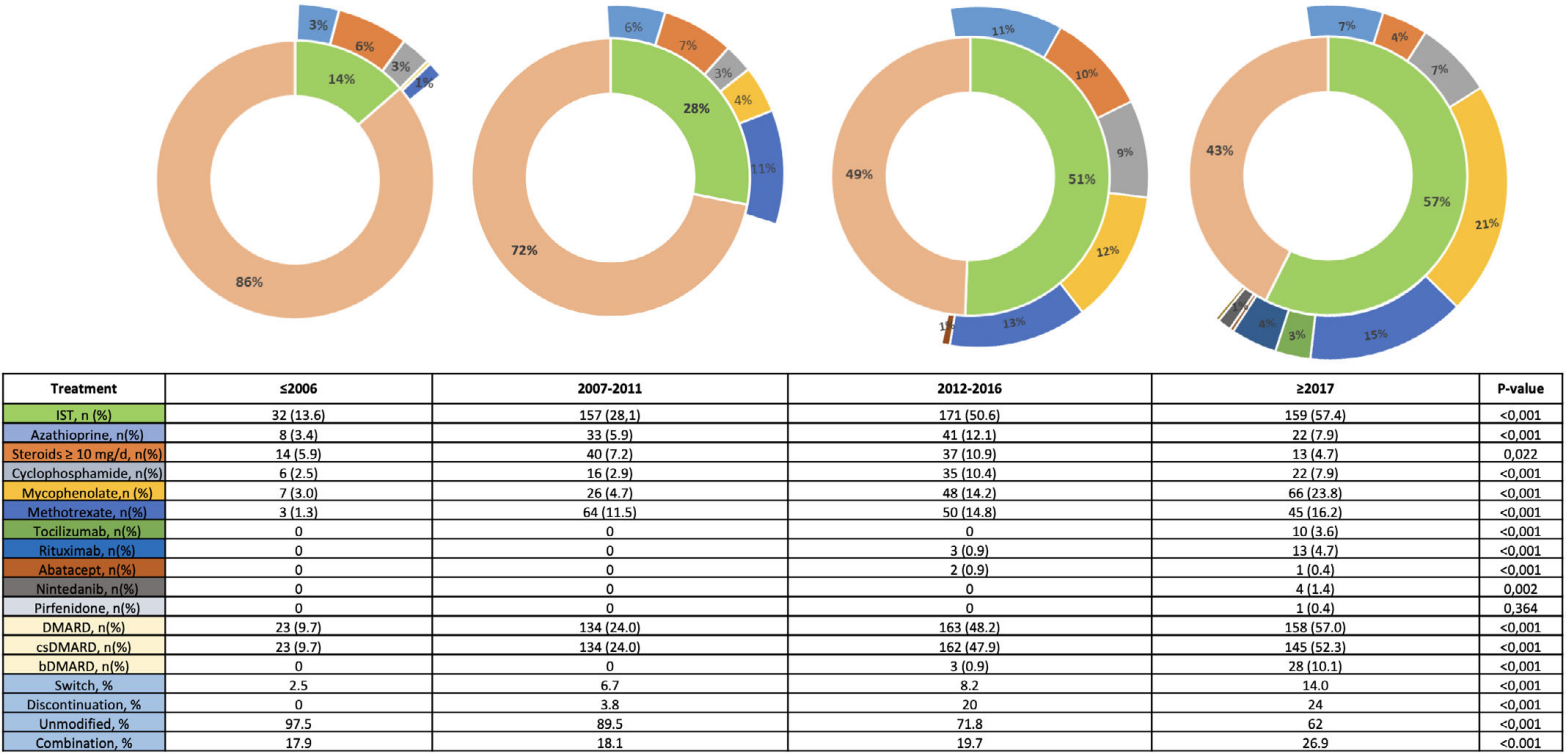
Changes of the treatment landscape of SSc‐ILD over time. bDMARD, biologic disease‐modifying antirheumatic drugs; csDMARD; conventional synthetic DMARD; IST, immunosuppressive therapy; SSc‐ILD, systemic sclerosis‐associated interstitial lung disease.

First, we wanted to assess whether treatment patterns changed over time. A total of 1,409 patients with SSc‐ILD were included across the four periods. The mean age at diagnosis was 54.7 ± 9.3 years, and 78.4% of the study population were women. The distribution of patients across the four treatment periods was as follows: (1) ≤2006: 236 patients (16.7%); (2) 2007–2011: 558 patients (39.6%); (3) 2012–2016: 338 patients (23.9%); and (4) ≥2017: 277 patients (19.7%). We found that the use of ISTs increased significantly, rising from 13.6% in the period 1, to 28.1% in period 2, then 50.6% in period 3, and peaking in period 4 with 57.4% (*P* < 0.001) (Figure 1). Antifibrotic therapies were first used in the contemporary period. Across the study periods, novel therapies were progressively introduced, leading to substantial changes in treatments used. MMF increased from 3% in the 2006 and earlier cohort to 24% in the 2017 and later cohort (*P* < 0.001), with 21% of treated patients receiving it as monotherapy. The use of CYC decreased from 18.7% among all ISTs in the 2006 and earlier cohort to 13.8% among all ISTs in the 2017 and later cohort (*P* < 0.001). Azathioprine decreased from 25% among ISTs in the 2006 and earlier cohort to 13.8% in the 2017 and later cohort (*P* < 0.001). Among csDMARDs, MMF was the most common treatment and was used by 29.6% of patients in the 2012 to 2016 cohort, increasing to 45.5% in the 2017 and later cohort (*P* < 0.001). Regarding biologics, RTX was the most used therapy, peaking at 46.4% among biologics in the 2017 and later cohort (see Figure 1).

Switching therapies increased from 2.5% in the 2006 and earlier cohort to 14% in the 2017 and later cohort (*P* < 0.001). The percentage of patients who discontinued therapies rose from 0% in the 2006 and earlier cohort to 24.0% in 2017 and later cohort (*P* < 0.001), whereas the proportion of patients with unmodified treatment decreased from 97.5% in the 2006 and earlier cohort to 62.0% in the 2017 and later cohort (*P* < 0.001). Last, the frequency of combination treatments increased from 17.9% in the 2006 and earlier cohort to 26.9% in the 2017 and later cohort (*P* < 0.001).

### The profiles of patients with SSc‐ILD over time

Next, we wanted to assess whether the characteristics of SSc‐ILD treated patients changed over time. Characteristics of the entire SSc‐ILD cohorts across the four time periods were similar except for statistically significant differences in median disease duration, an increase in sine‐scleroderma patients, and a significant reduction in patients with myositis (Supplementary Table 1). When analyzing the characteristics of treated patients with SSc‐ILD across the last three periods, excluding period 1 because of the limited number of treated cases (n = 32), we observed a significant increase in the median age at treatment initiation from period 2 to period 4 (Table 1). Additionally, patients in period 4 presented lower %pFVC and %pDLco values. A progressive reduction in the prevalence of concomitant inflammatory arthritis was also noted, decreasing from 23.1% in period 2 to 17.9% in period 3 and 12.2% in period 4 (*P* = 0.042). Similarly, the prevalence of myositis declined from 32.0% in period 2 to 17.5% in period 3 and 14.5% in period 4 (*P* = 0.038). An increasing prevalence of ARA positivity was observed, rising from 1.9% in period 2 to 2.4% in period 3 and 8.7% in period 4 (*P* = 0.038) (Table 1). Including treated patients from period 1, we could also confirm a significant increase in the median age at treatment initiation from period 1 to period 4, a significant increase in the percentage of male patients treated (from 9.3% in period 1 to 28.3% in period 4; *P* = 0.028) and a decrease in the rate of patients with concomitant myositis (from 29.0% in period 1 to 14.5% in period 4; *P* = 0.038) (see Supplementary Table 2). The overall rate of deaths (within 5 years since the first evaluation) among treated patients belonging to the four periods was as follows: 1 (3.1%) in period 1; 7 (4.5%) in period 2; 11 (6.4%) in period 3; and 8 (5.0%) in period 4 (*P* = 0.803). Conversely, lung transplantation rates among the same cohorts were as follows: 1 of 32 (3.1%) in period 1; 3 of 157 (1.9%) in period 2; 3 of 171 (1.7%) in period 3; and 1 of 159 (0.6%) in period 4 (*P* = 0.660).

**Table 1:** Demographic and clinical features of patients with SSc‐ILD treated at first‐time assessment across the three periods (total 487 patients)*

Time period	2007–2011	2012–2016	≥2017	*P* value
	(N = 157)	(N = 171)	(N = 159)	
Age, mean (SD), years	52.1 (12.6)	52.1 (12.8)	55.8 (11.8)	**0.010**
Male, n (%)	34 (21.7)	41 (24.0)	45 (28.3)	0.387
Disease duration, median (IQR), months	55.0 (28.2–115.5)	55.5 (25.0–98.0)	53.5 (18.0–95.0)	0.562
%pDLco, mean (SD)	62.5 (18.6)	55.1 (18.6)	59.4 (19.1)	**0.003**
%pFVC, mean (SD)	87.7 (19.9)	80.5 (19.7)	81.4 (21.0)	**0.006**
Known smoking status	18	122	128	0.258
Ever smoker, n (%)	3 (16.6)	43 (35.2)	47 (36.7)	
Known skin involvement status, n (%)	156	162	79	
Sine scleroderma	3 (1.9)	7 (4.3)	6 (7.6)	0.111
Limited cutaneous	61 (39.1)	69 (42.6)	27 (34.2)	0.456
Diffuse cutaneous	92 (59.0)	86 (53.1)	46 (58.2)	0.549
Stomach symptoms, n (%)	39/156 (25.0)	41/167 (24.5)	35/156 (22.4)	0.876
Intestinal symptoms, n (%)	25/155 (16.7)	27/167 (16.2)	33/156 (21.1)	0.479
Scleroderma renal crisis, n (%)	3 (1.9)	2 (1.2)	1 (0.6)	0.615
Digital ulcers, n (%)	62/138 (44.9)	64/144 (43.7)	69/155 (44.5)	0.918
Inflammatory arthritis, n (%)	36/156 (23.1)	30/168 (17.9)	19/156 (12.2)	**0.042**
Myositis, n (%)	40/156 (32.0)	29/166 (17.5)	23/158 (14.5)	**0.038**
PAH, n (%)	0/29 (0)	1/44 (2.3)	0/33 (0)	0.999
Known antibodies status, n (%)	155	161	148	
Anticentromere	18 (11.6)	13 (8.1)	8 (5.4)	0.192
Antitopoisomerase	95 (61.3)	104 (64.5)	94 (63.5)	0.826
Anti‐RNA‐polymerase III	3 (1.9)	4 (2.4)	13 (8.7)	**0.038**

* %pDLco, % predicted diffusing capacity of the lungs for carbon monoxide; %pFVC, %‐predicted forced vital capacity; IQR, interquartile range; PAH, pulmonary arterial hypertension; SSc‐ILD, systemic sclerosis‐associated interstitial lung disease.

### Impact of immunosuppressive treatment patterns on ILD progression

**Figure 2:**
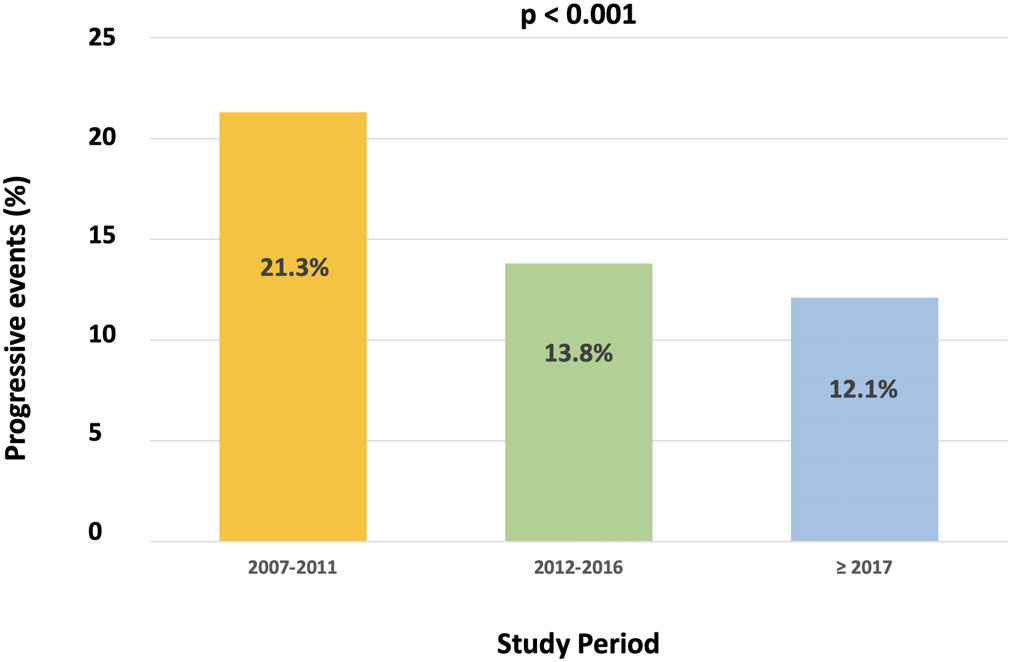
Number of progressive events in patients with SSc‐ILD treated with immunosuppressive and/or antifibrotic drugs among the three cohorts segregated by time. SSc‐ILD, systemic sclerosis‐associated interstitial lung disease. Color figure can be viewed in the online issue, which is available at http://onlinelibrary.wiley.com/doi/10.1002/art.70043/abstract.

Last, we analyzed the impact of IST and antifibrotic therapies across the different time periods in patients with SSc‐ILD on ILD progression. Period 1 was excluded from the analyses given the low number of treated patients. Over a follow‐up period of three years, the number of progressive events decreased significantly across the periods; from 115 of 540 (21.3%) in period 2; to 146 of 1061 (13.8%) in period 3; and 160 of 1320 (12.1%) in period 4 (*P* < 0.001), respectively (Figure 2). We conducted the same analyses in ATA‐positive patients, enriching for a more severe SSc‐ILD cohort. ATA‐positive patients represented 56% of the SSc‐ILD population across the four periods (136, 311, 185, and 154 patients in periods 1 to 4, respectively). Even in this subgroup, the proportion of progressive events decreased significantly over time, from 73 of 318 (23.0%) in period 2 to 84 of 563 (14.9%) in period 3 and 83 of 654 (12.7%) in period 4 (*P* ≤ 0.001).

We then analyzed what modifications were made after the first progressive event among the three cohorts in patients already on ISTs and/or antifibrotic therapy, and we observed that there was a significant increase in combination therapies and switching therapies from period 2 to period 4 (see Table 2).

**Table 2:** Treatment modifications after the first progressive event in patients on immunosuppressive and/or antifibrotic therapy belonging to period 2: 2007–2011, period 3: 2012–2016, and period 4: ≥2017*

Treatment modification	Period 2 (N = 115)	Period 3 (N = 146)	Period 4 (N = 160)	*P* value
Switch, n (%)	11 (9.6)	13 (8.9)	27 (16.9)	**0.049**
Discontinuation, n (%)	6 (5.2)	10 (6.8)	9 (5.6)	0.870
Retained same drug, n (%)	98 (85.2)	121 (82.9)	124 (77.5)	0.238
Combination, n (%)	24 (20.9)	27 (18.5)	52 (32.5)	**0.011**

* Bolded values significant *P* < 0.05.

### Current treatment patterns

**Figure 3:**
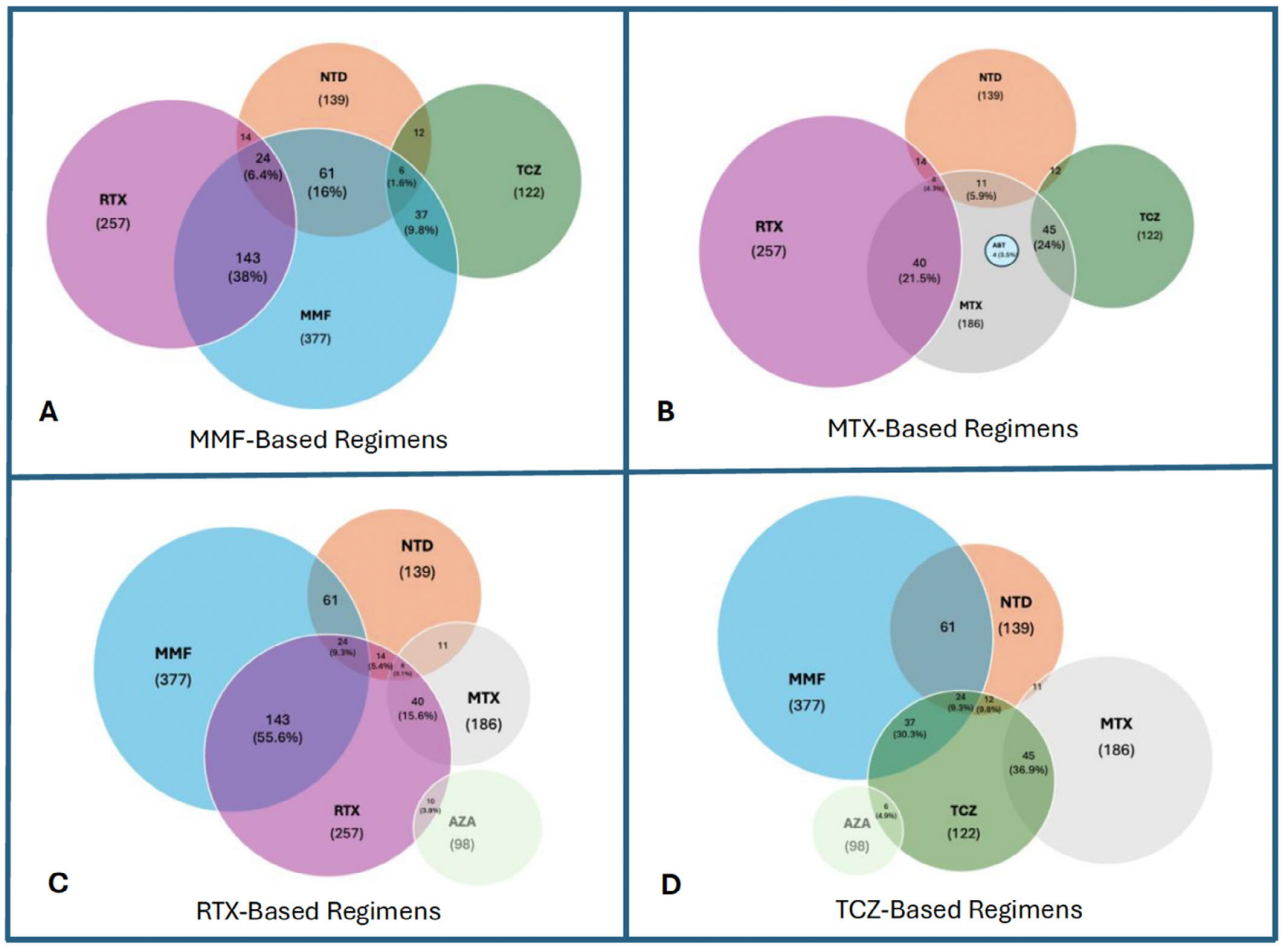
Treatment courses in patients treated in year 2017 or after according to the DMARD‐based regimen: (A) MMF‐based courses; (B) MTX‐based courses; (C) RTX‐based courses; and (D) TCZ‐based courses. AZA, azathioprine; DMARD, disease‐modifying antirheumatic drug; MMF, mycophenolate mofetil; MTX, methotrexate; NTD, nintedanib; RTX, rituximab; TCZ, tocilizumab. Color figure can be viewed in the online issue, which is available at http://onlinelibrary.wiley.com/doi/10.1002/art.70043/abstract.

To characterize the treatment pattern of patients with SSc‐ILD in the contemporary cohort, we performed a detailed analysis of patients belonging to period 4 (≥2017). Among 277 patients, 118 were untreated, and among the treated, a total of 377 MMF‐based treatment courses were identified. MMF was used as monotherapy in 166 (44%) treatment courses, whereas it was combined with RTX in 143 (38%) courses, with nintedanib in 61 (16%) courses and with tocilizumab in 37 (10%) courses (Figure 3A). In 24 (6.4%) courses MMF was used in triple combination with RTX and nintedanib. Methotrexate was used in monotherapy in 86 (46%) courses; in combination with tocilizumab in 45 cases (24%); with RTX in 40 (21.5%); and with nintedanib in 11 (5.9%) courses. In eight courses (4.3%) methotrexate was used in triple combination (Figure 3B). Rituximab (n = 257) was in the majority of cases used in double combination (175 cases, 68%), with MMF being the most common and only in 50 courses (19%) as monotherapy. In 32 (12%) regimens RTX was used in triple combination (Figure 3C). Last, tocilizumab‐based courses accounted for a total of 122 cases with 22 (18%) used as monotherapy. The majority included combination therapies with MTX (45 cases, 36.9%). Triple combination was observed in 24 (9.3%) cases (Figure 3D).

**Figure 4:**
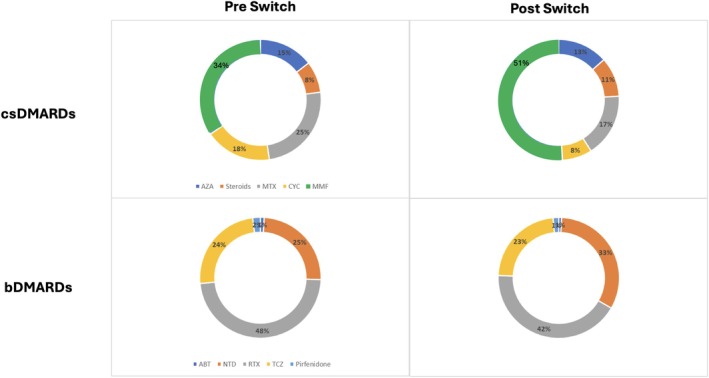
The use of csDMARDs and bDMARDs in patients with SSc‐ILD switching therapies in the contemporary period. ABT, abatacept; AZA, azathioprine; bDMARDs, biologic synthetic disease‐modifying antirheumatic drugs; cDMARDs, conventional synthetic DMARDs; CYC, cyclophosphamide; MMF, mycophenolate mofetil; MTX, methotrexate; NTD, nintedanib; RTX, rituximab; SSc‐ILD, systemic sclerosis‐associated interstitial lung disease; TCZ, tocilizumab. Color figure can be viewed in the online issue, which is available at http://onlinelibrary.wiley.com/doi/10.1002/art.70043/abstract.

A total of 14% treatments were switched in the contemporary cohort. After switching, a decrease in csDMARD therapies was observed (pre‐switch use of 90.1% to post‐switch use of 85.5%), whereas an increase in bDMARD therapies was observed from 27% to 51%. The type of csDMARD and bDMARD post switching did not significantly change, with MMF being the most common csDMARD, and RTX the most common bDMARD (see Figure 4).

### The impact of clinical characteristics on treatment patterns in the contemporary cohort

We wanted to assess which clinical characteristics influenced treatment choices in the contemporary cohort, so we conducted univariable and multivariable logistic regression analyses (Supplementary Tables 3–6). We identified that shorter disease duration was significantly associated with IST and antifibrotic therapy initiation at the first evaluation (OR 0.991, 95% CI 0.987–0.996; *P* < 0.001). Additionally, the presence of concomitant myositis was strongly linked to IST and antifibrotic therapy initiation (OR 9.934, 95% CI 1.936–51.764; *P* = 0.006). The likelihood of switching treatments was significantly higher in patients with a higher mRSS (OR 1.031, 95% CI 1.002–1.062; *P* = 0.035) and in patients with arthritis (OR 3.034, 95% CI 1.551–5.936; *P* = 0.001). No clinical feature was found to be significantly associated with treatment discontinuation. Throughout the study period, the use of combination therapy was associated with younger age (OR 0.968, 95% CI 0.953–0.985; *P* < 0.001), higher dyspnea class (OR 1.557, 95% CI 1.189–2.039; *P* = 0.001), and inflammatory arthritis (OR 2.560, 95% CI 1.354–4.840; *P* = 0.004).

## DISCUSSION

Our study provides novel insights into the change of IST and antifibrotic treatment patterns for patients with SSc‐ILD over the past decades using a large and multicenter real‐world SSc‐ILD cohort from the EUSTAR database. Our findings highlight a substantial shift in clinical practice, showing the integration of new therapeutic agents and evolving treatment strategies that are driven by increasing evidence and expanded therapeutic options.

The first key observation is the significant increase of patients treated with IST and/or antifibrotic treatment since their first evaluation, with a peak reached in the contemporary cohort, in which more than 50% of patients are treated. This trend was already observed in a 2018 EUSTAR study,^21^ which showed that although the majority (>70%) of patients with SSc‐ILD receive IST at some point, its use was still not widespread, especially in early patients. Watchful waiting and close monitoring were commonly employed in the early stages, with IST initiation typically reserved for patients with moderate lung function impairment. In the present study, we further explored this aspect by analyzing patients at their first evaluation across four distinct time periods, demonstrating how the watchful waiting strategy has been progressively replaced by earlier treatment initiation. Nevertheless, 43% of patients with SSc‐ILD still do not receive IST at their first evaluation.

Although the overall patient profile did not change dramatically over time, we observed a progressive refinement in the features of patients initiated on IST and/or antifibrotic therapies based on ILD characteristics in the contemporary cohort. Older patients with SSc‐ILD were more frequently started on IST and/or antifibrotic therapy at their first evaluation. At the same time, extrapulmonary involvement—such as inflammatory arthritis and myositis, which may have previously driven treatment decisions—was significantly less represented in the contemporary cohort. Similarly, the prevalence of sine‐scleroderma patients within the overall SSc‐ILD cohort was lower in more recent diagnoses. This shift reflects a growing understanding of SSc‐ILD.^22^ This has also been incorporated in the design of the most recent clinical trials focusing on ILD, in which patients regardless of their cutaneous subset are included, such as in the SENSCIS and FIBRONEED‐ILD trials.^11^, ^23^, ^24^

We also identified an increasing complexity of treatment regimens, with physicians not only integrating novel therapies such as RTX, tocilizumab, and nintedanib but also increasingly using combination therapies and treatment‐switching strategies. Although minimal variability in visit frequency or completeness of date fields across centers cannot be excluded, the use of predefined structured variables ensured consistency in treatment classification throughout the registry. We observed limited use of nintedanib. This aligns with the study window (2017–2022) of contemporary patients, which overlaps only the initial post‐approval years in Europe and the United States, and therefore more likely reflects recent adoption than underuse in current care.

Previous EUSTAR studies partly highlighted that the use of advanced therapies were incorporated in the clinical practice of EUSTAR centers even in combination with other csDMARDs.^25^, ^26^, ^27^, ^28^ Nonetheless, our study for the first time highlights how these changes have impacted the progression of patients with SSc‐ILD and how treatment strategies have evolved over time.

This trend reflects a more tailored, patient‐centered approach to SSc‐ILD management, allowing for dynamic treatment adjustments based on individual responses. Moreover, our analysis highlights that combination therapies have been preferentially used in younger patients with a higher dyspnea class and concomitant presence of inflammatory arthritis, emphasizing the growing clinical need for intensified therapeutic regimens in young patients who already have pulmonary disability due to the underlying ILD. The increasing use of these strategies underscores the significant unmet need for more evidence for sophisticated and combined approaches, including upfront and sequential combination strategies, to overcome therapeutic limitations and enhance disease control.^29^ This appears to be particularly relevant for patients who have already received first‐line IST and/or antifibrotic therapy. In those who progressed despite treatment, we observed a significant increase over time in the use of combination and switching strategies. This underscores how physicians are increasingly and proactively adapting treatment for nonresponsive patients, integrating newly approved drugs and/or layering them onto existing regimens.^16^ However, the evidence supporting combination treatment regimens—when to modify them and which patients are best suited for such an approach—remains limited. High‐quality data are available only from the SENSCIS trial,^11^ which evaluated the efficacy and safety of a single combination (MMF and nintedanib).^30^ In contrast, the use of nintedanib in combination with other drugs or in triple therapy regimens has been reported only in retrospective studies.^31^, ^32^ Furthermore, data on other agents, such as RTX and tocilizumab, are even more scarce^32^, ^33^, ^34^, ^35^, ^36^ because major clinical trials did not allow concurrent ISTs.^16^

Our findings also demonstrate that early IST initiation has become standard practice in SSc‐ILD management. MMF has emerged as a cornerstone drug, serving as the backbone for many combination regimens with biologics and nintedanib. As shorter disease duration emerged as a predictor for IST and/or antifibrotic therapy initiation at the first evaluation in contemporary patients, this has important implications for the design of future clinical trials. Short disease duration is indeed an inclusion criterion in clinical trials, and given that the majority of patients with SSc‐ILD receive IST at their first evaluation, trial design must reflect this reality, ensuring that investigational therapies are evaluated in the context of background treatment.^37^ Furthermore, to align with clinical practice and our findings, it is essential to acknowledge the use of various background therapies beyond MMF and nintedanib, including RTX and tocilizumab. These agents are now part of the approved treatment landscape for SSc‐ILD and should be considered as background therapies and/or control arms in future trials.^38^ Even in the most recent SSc‐ILD trials, many of these drugs remain prohibited, with washout periods often required. This may contribute to low patient enrollment rates and an increased number of screening failures.^17^

This crucial aspect should also be interpreted within the broader context that, despite the apparent benefits of evolving treatment strategies, prognosis for patients with SSc‐ILD remains unsatisfactory. Although our findings suggest that broader use of treatment may have contributed to reduced short‐term ILD progression rates, a substantial proportion of patients still experience disease worsening. This has been observed not only in our analysis but also in previous studies conducted within the EUSTAR network in recent years.^39^ They report that 30% of patients experience disease progression annually, regardless of treatment status. Furthermore, in our study, we found that the proportion of patients experiencing progressive events has remained relatively stable, with rates of approximately 14% in period 3 and 12% in period 4. This underscores the urgent need for more effective therapeutic options and treatment regimens, especially because even a 5% decrease in the %pFVC in one year has been shown to be associated with increased mortality.^40^

This study has some limitations. First, the lack of serial HRCT data and the earlier detection of milder disease and/or a more treatment‐responsive phenotype over more recent years could contribute to the observed improvements and prevent a direct assessment of radiologic ILD progression, limiting our ability to correlate treatment strategies with imaging‐based outcomes.^41^ Second, although we examined IST and antifibrotic treatment patterns, we did not assess whether treatment initiation was solely driven by ILD indication or by other SSc‐related manifestations. Third, safety data, including adverse events, were not analyzed because side effects are not systematically recorded in the EUSTAR database. Finally, our study does not account for patient adherence or compliance with given therapies, which could influence real‐world treatment effectiveness. These limitations highlight the need for future research integrating imaging data, safety outcomes, and adherence metrics to provide a more comprehensive understanding of IST and antifibrotic impact in SSc‐ILD. Nonetheless, the ability of EUSTAR to capture real‐life practice in many patients with a temporal continuum represents a major strength of our work. It paves the way and is instrumental for the design of future trials.

In conclusion, this study reveals a clear change in the management of SSc‐ILD, which is nowadays characterized by treatment initiation at a patient's first evaluation in most patients with SSc‐ILD, the widespread use of ILD specific treatment options, and the increasing use of combination and switch strategies. As treatment paradigms continue to evolve, future research must integrate these real‐world patterns into clinical trial design and explore novel therapeutic targets to further improve patient outcomes. Despite significant progress, the prognosis for SSc‐ILD remains suboptimal, emphasizing the ongoing need for innovative treatment approaches and refined patient stratification strategies.

## AUTHOR CONTRIBUTIONS

All authors contributed to at least one of the following manuscript preparation roles: conceptualization AND/OR methodology, software, investigation, formal analysis, data curation, visualization, and validation AND drafting or reviewing/editing the final draft. As corresponding author, Dr Hoffmann‐Vold confirms that all authors have provided the final approval of the version to be published and takes responsibility for the affirmations regarding article submission (eg, not under consideration by another journal), the integrity of the data presented, and the statements regarding compliance with institutional review board/Declaration of Helsinki requirements.

## Supporting information


**Disclosure form**.

**Table S1:** Demographic and clinical features of SSc‐ILD patients at first‐time assessment across the 4 periods segregated by period.
**Table S2:** Demographic and clinical features of SSc‐ILD patients treated at first‐time assessment across the 4 periods (total 519 patients).
**Table S3:** Univariable and Multivariable Logistic Regression analysis for immunosuppressive and anti‐fibrotic therapy introduction in SSc‐ILD patients belonging to cohort 4 (>= 2017).
**Table S4:** Univariable and Multivariable Logistic Regression analysis for combination therapy in SSc‐ILD patients belonging to cohort 4 (>= 2017).
**Table S5:** Univariable and Multivariable Logistic Regression analysis for therapy stop in SSc‐ILD patients belonging to cohort 4 (>= 2017).
**Table S6:** Univariable and Multivariable Logistic Regression analysis for therapy switch in SSc‐ILD patients belonging to cohort 4 (>= 2017).
