# Supplementary material for: Impact of Evolving Treatment Patterns on Interstitial Lung Disease Progression in Systemic Sclerosis Using the European Scleroderma Trials and Research Database
Source: Arthritis Rheumatol. 2026 Mar 29;78(8):1755–64. doi: 10.1002/art.70043 (PMC13430113; doi:10.1002/art.70043)
Supplement: Supplementary file 2 — Table S1: Demographic and clinical features of SSc‐ILD patients at first‐time assessment across the 4 periods segregated by period. Table S2: Demographic and clinical features of SSc‐ILD patients treated at first‐time assessment across the 4 periods (total 519 patients). Table S3: Univariable and Multivariable Logistic Regression analysis for immunosuppressive and anti‐fibrotic therapy introduction in SSc‐ILD patients belonging to cohort 4 (>= 2017). Table S4: Univariable and Multivariable Logistic Regression analysis for combination therapy in SSc‐ILD patients belonging to cohort 4 (>= 2017). Table S5: Univariable and Multivariable Logistic Regression analysis for therapy stop in SSc‐ILD patients belonging to cohort 4 (>= 2017). Table S6: Univariable and Multivariable Logistic Regression analysis for therapy switch in SSc‐ILD patients belonging to cohort 4 (>= 2017). [file ART-78-1755-s002.docx]

**Table 1s**. Demographic and clinical features of SSc-ILD patients at first-time assessment across the 4 periods segregated by period.

| **Time Period** | **≤ 2006 (n = 236)** | **2007-2011 (n = 558)** | **2012 – 2016 (n = 338)** | **≥ 2017 (n = 277)** | **p-value** |
| --- | --- | --- | --- | --- | --- |
| Age (SD) | 56.1 (12.3) | 55.0 (13.2) | 54.4 (13.2) | 56.8 (11.8) | 0.495 |
| Male (%) | 37 (15.7) | 122 (21.9) | 65 (19.2) | 61 (22.0) | 0.188 |
| Follow-up, months | 86 (41.5 – 129) | 77 (45 – 120) | 50 (34 – 72) | 29 (24 – 39.5) | **<0.001** |
| Disease Duration (months), median (IQR) | 83.0 (44.2 – 151.5) | 61.5 (30.0 – 128.2) | 66.0 (28.0 – 140.5) | 61.0 (22.2 – 138.5) | **<0.001** |
| Patients with 3-year follow-up on IST (%) | NA | 98 (17.6) | 106 (31.3) | 93 (33.6) | 0.683 |
| %pDLCO (SD) | 62.4 (23.6) | 61.6 (19.1) | 58.2 (19.4) | 59.6 (19.8) | 0.240 |
| %pFVC (SD) | 85.0 (21.7) | 87.6 (21.3) | 84.1 (20.1) | 82.5 (21.0) | 0.073 |
| Known smoking status  Ever smoker (%) | 11  3 (27.3) | 50  15 (30.0) | 215  70 (32.6) | 233  86 (36.9) | 0.685 |
| *Known skin involvement status*  Sine scleroderma (%)  Limited cutaneous (%)  Diffuse cutaneous (%) | 236  1 (0.4)  111 (47.0)  124 (52.5) | 557  4 (0.7)  265 (47.6)  288 (51.7) | 307  12 (3.9)  151 (49.2)  144 (46.9) | 119  13 (10.9)  48 (40.3)  58 (48.7) | **<0.001**  0.429  0.486 |
| Esophageal symptoms (%)  Stomach symptoms (%)  Intestinal symptoms (%)  Scleroderma Renal Crisis (%)  Digital Ulcers (%)  Inflammatory Arthritis (%)  Myositis (%)  PAH (%) | 165/235 (70.2)  60/235 (25.5)  62/236 (26.3)  2 (0.9)  13/25 (52.0)  39 (16.7)  60 (25.6)  0/1 (0) | 372/557 (66.8)  137/555 (24.7)  104/555 (18.7)  8 (1.4)  116/254 (45.7)  89 (16.0)  131 (23.6)  3/76 (3.9) | 213/334 (63.8)  77/333 (23.1)  59/333 (17.7)  3 (0.9)  93/202 (46.0)  47 (14.1)  50 (15.3)  1/48 (2.1) | 175/271 (64.6)  52/273 (19.0)  57/273 (20.9)  2 (0.7)  117/263 (44.5)  28 (10.3)  33 (12.1)  4/125 (3.2) | 0.395  0.254  0.064  0.839  0.769  0.117  **<0.001**  0.668 |
| *Known antibodies status*  Anti-centromere (%)  Anti-topoisomerase (%)  Anti-RNA-polymeraseIII (%) | 234  33 (14.1)  136 (58.1)  1 (0.4) | 547  77 (14.1)  311 (56.8)  10 (1.8) | 307  41 (13.4)  185 (60.2)  11 (3.5) | 243  26 (10.7)  154 (63.3)  19 (7.8) | 0.604  0.703  0.137 |

**Table 2s.** Demographic and clinical features of SSc-ILD patients treated at first-time assessment across the 4 periods (total 519 patients).

| **Time Period** | **≤2006 (n =32)** | **2007-2011 (n = 157)** | **2012 – 2016 (n = 171)** | **≥ 2017 (n = 159)** | **p-value** |
| --- | --- | --- | --- | --- | --- |
| Age (SD) | 52.9 (12.5) | 52.1 (12.6) | 52.1 (12.8) | 55.8 (11.8) | **0.026** |
| Male (%) | 3 (9.3) | 34 (21.7) | 41 (24.0) | 45 (28.3) | **0.028** |
| Disease Duration (months), median (IQR) | 63.0 (32.0 – 94.0) | 55.0 (28.2 – 115.5) | 55.5 (25.0 – 98.0) | 53.5 (18.0 – 95.0) | 0.655 |
| %pDLCO (SD) | 59.7 (16.8) | 62.5 (18.6) | 55.1 (18.6) | 59.4 (19.1) | **0.007** |
| %pFVC (SD) | 77.4 (16.0) | 87.7 (19.9) | 80.5 (19.7) | 81.4 (21.0) | **0.014** |
| Known smoking status  Ever smoker (%) | 2  1 (33) | 18  3 (16.6) | 122  43 (35.2) | 128  47 (36.7) | 0.148 |
| *Known skin involvement status*  Sine scleroderma (%)  Limited cutaneous (%)  Diffuse cutaneous (%) | 32  0 (0)  9 (28.1)  23 (71.8) | 156  3 (1.9)  61 (39.1)  92 (59.0) | 162  7 (4.3)  69 (42.6)  86 (53.1) | 79  6 (7.6)  27 (34.2)  46 (58.2) | 0.135  0.370  0.250 |
| Esophageal symptoms (%)  Stomach symptoms (%)  Intestinal symptoms (%)  Scleroderma Renal Crisis (%)  Digital Ulcers (%)  Inflammatory Arthritis (%)  Myositis (%)  PAH (%) | 20/32 (62.5)  7/32 (21.9)  9/32 (28.1)  1/32 (3.1)  14/30 (46.7)  8/32 (25.0)  9 /31 (29.0)  0/24 | 99/157 (63.1)  39/156 (25.0)  25/155 (16.7)  3 (1.9)  62/138 (44.9)  36/156 (23.1)  40/156 (32.0)  0/29 (0) | 111/169 (65.7)  41/167 (24.5)  27/167 (16.2)  2 (1.2)  64/144 (43.7)  30/168 (17.9)  29/166 (17.5)  1/44 (2.3) | 106/157 (67.5)  35/156 (22.4)  33/156 (21.1)  1 (0.6)  69/155 (44.5)  19/156 (12.2)  23/158 (14.5)  0/33 (0) | 0.840  0.955  0.308  0.393  0.865  0.053  **0.038**  0.999 |
| *Known antibodies status*  Anti-centromere (%)  Anti-topoisomerase (%)  Anti-RNA-polymeraseIII (%) | 32  2 (6.2)  22 (68.7)  1 (3.1) | 155  18 (11.6)  95 (61.3)  3 (1.9) | 161  13 (8.1)  104 (64.5)  4 (2.4) | 148  8 (5.4)  94 (63.5)  13 (8.7) | 0.335  0.781  0.070 |

**Table 3s**. Univariable and Multivariable Logistic Regression analysis for immunosuppressive and anti-fibrotic therapy introduction in SSc-ILD patients belonging to cohort 4 (>= 2017).

Total number of patients starting immunosuppressive treatment in period 4 at their first evaluation: 629/1110.

Availability of data: Sex (1110), Age (1110), disease duration (900/210),%pDLCO (974/136), %pFVC (1093/17), dyspnoea class (363/747), Disease subset (379/731), mRSS (957/153), esophageal symptoms (1075/35), inflammatory arthritis (1065/45), myositis (1062/48), autoantibodies (956/154), CRP-elevation (363/747).

| **Variable** | **Univariable Analysis** | **P-value** | **Multivariable Analysis** | **P-value** |
| --- | --- | --- | --- | --- |
| Male Sex | 1.220 (0.902 – 1.650) | 0.197 |  |  |
| Age | 0.977 (0.968 – 0.986) | <0.001 | 0.992 (0.962 – 1.024) | 0.624 |
| Disease Duration | 0.997 (0.996 – 0.999) | <0.001 | 0.991 (0.987 – 0.996) | <0.001 |
| %pDLCO | 0.995 (0.989 – 1.002) | 0.138 |  |  |
| %pFVC | 0.988 (0.982 – 0.994) | <0.001 | 0.995 (0.978 – 1.013) | 0.590 |
| Dyspnoea Class | 1.114 (0.809 – 1.532) | 0.509 |  |  |
| DcSSc | 2.136 (1.385 – 3.294) | <0.001 | 1.340 (0.571 – 3.144) | 0.502 |
| mRSS | 1.044 (1.026 – 1.061) | <0.001 | 1.011 (0.966 – 1.059) | 0.633 |
| Esophageal symptoms | 1.259 (0.983 – 1.614) | 0.069 | 0.779 (0.253 – 2.379) | 0.664 |
| Inflammatory Arthrits | 1.811 (1.178 – 2.783) | 0.007 | 1.147 (0.278 – 4.734) | 0.849 |
| Myositis | 2.162 (1.412 – 3.311) | <0.001 | 9.934 (1.906 – 51.764) | 0.006 |
| ACA | 0.449 (0.310 – 0.650) | <0.001 | 0.562 (0.175 – 1.806) | 0.291 |
| ATA | 1.397 (1.077 – 1.811) | 0.012 | 1.447 (0.714 – 2.931) | 0.305 |
| ARA | 1.732 (1.001 – 2.998) | 0.050 | 4.017 (0.927 – 17.406) | 0.133 |
| CRP-elevation | 0.929 (0.578 – 1.491) | 0.759 |  |  |

**Table 4s**. Univariable and Multivariable Logistic Regression analysis for combination therapy in SSc-ILD patients belonging to cohort 4 (>= 2017).

Total number of therapy courses with combination therapy in period 4: 787/3419.

Availability of data: Sex (3419), Age (3419), disease duration (3038/381),%pDLCO (2981/438), %pFVC (3362/57), dyspnoea class (1261/2158), Disease subset (1269/2150), mRSS (3100/319), esophageal symptoms (3340/79), inflammatory arthritis (3267/152), myositis (3254/165), autoantibodies (2739/680), CRP-elevation (1196/2223).

| Variable | Univariable Analysis | P-value | Multivariable Analysis | P-value |
| --- | --- | --- | --- | --- |
| Male Sex | 1.622 (1.349 – 1.949) | <0001 | 1.372 (0.916 – 2.055) | 0.125 |
| Age | 0.984 (0.978 – 0.991) | <0001 | 0.968 (0.953 – 0.985) | <0.001 |
| Disease Duration | 0.998 (0.997 – 0.999) | <0.001 | 0.999 (0.997 – 1.001) | 0.590 |
| %pDLCO | 0.979 (0.975 – 0.984) | <0.001 | 0.993 (0.982 – 1.004) | 0.221 |
| %pFVC | 0.985 (0.982 – 0.989) | <0.001 | 1.004(0.992 – 1.015) | 0.514 |
| Dyspnoea Class | 1.604 (1.358 – 1.895) | <0.001 | 1.557 (1.189 – 2.039) | 0.001 |
| DcSSc | 1.805 (1.355 – 2.403) | <0.001 | 1.137 (0.767 – 1.685) | 0.522 |
| mRSS | 1.015 (1.005 – 1.025) | 0.003 | 1.004 (0.981 – 1.028) | 0.746 |
| Esophageal symtoms | 1048 (0.889 – 1.235) | 0.580 |  |  |
| Inflammatory Arthrits | 1.848 (1.444 – 2.366) | <0.011 | 2.560 (1.354 – 4.840) | 0.004 |
| Myositis | 1.344 (1.070 – 1.688) | 0.011 | 0.894 (0.490 – 1.632) | 0.715 |
| ACA | 0.0725 (0.523 – 1.005) | 0.054 | 0.988 (0.481 – 2.029) | 0.974 |
| ATA | 0.946 (0.778 – 1.136) | 0.551 |  |  |
| ARA | 0.754 (0.500 – 1.137) | 0.177 |  |  |
| CRP-elevation | 1.186 (0.865 – 1.626) | 0.288 |  |  |

**Table 5s**. Univariable and Multivariable Logistic Regression analysis for therapy stop in SSc-ILD patients belonging to cohort 4 (>= 2017).

Total number of therapy courses stopped: 151/3419

| Variable | Univariable Analysis | P-value | Multivariable Analysis | P-value |
| --- | --- | --- | --- | --- |
| Male Sex | 0.830 (0.534 – 1.292) | 0.410 |  |  |
| Age | 0.996 (0.984 – 1.009) | 0.576 |  |  |
| Disease Duration | 1.000 (0.998 – 1.001) | 0.717 |  |  |
| %pDLCO | 0.998 (0.989 – 1.007) | 0.680 |  |  |
| %pFVC | 1.002 (0.995 – 1.009) | 0.596 |  |  |
| Dyspnoea Class | 0.857 (0.608 – 1.207) | 0.377 |  |  |
| DcSSc | 1.620 (0.992 – 2.646) | 0.054 | 1.321 (0.736 – 2.368) | 0.351 |
| mRSS | 1.034 (1.1015 – 1.054) | <0.001 | 1.032 (0.999 – 1.066) | 0.058 |
| Esophageal symtoms | 0.808 (0.581 – 1.123) | 0.205 |  |  |
| Inflammatory Arthrits | 0.995 (0.534 – 1.857) | 0.989 |  |  |
| Myositis | 0.876 (0.501 – 1.533) | 0.644 |  |  |
| ACA | 0.938 (0.531 – 1.657) | 0.825 |  |  |
| ATA | 1.172 (0.783 – 1.755) | 0.440 |  |  |
| ARA | 0.505 (0.158 – 1.613) | 0.249 |  |  |
| CRP-elevation | 0.779 (0.433 – 1.402) | 0.405 |  |  |

**Table 6s**. Univariable and Multivariable Logistic Regression analysis for therapy switch in SSc-ILD patients belonging to cohort 4 (>= 2017).

Total number of therapy courses switched: 453/3419

| Variable | Univariable Analysis | P-value | Multivariable Analysis | P-value |
| --- | --- | --- | --- | --- |
| Male Sex | 1.399 (1.114 – 1.757) | 0.004 | 1.159 (0.670 – 2.004) | 0.597 |
| Age | 0.993 (0.986 – 1.001) | 0.103 |  |  |
| Disease Duration | 0.998 (0.997 – 0.999) | 0.008 | 0.998 (0.995 – 1.000) | 0.070 |
| %pDLCO | 0.981 (0.976 – 0.987) | <0.001 | 0.986 (0.969 – 1.004) | 0.135 |
| %pFVC | 0.989 (0.984 – 0.993) | <0.001 | 0.994 (0.982 – 1.007) | 0.378 |
| Dyspnoea Class | 1.427 (1.142 – 1.785) | <0.001 | 1.239 (0.892 – 1.723) | 0.379 |
| DcSSc | 1.656 (1.147 – 2.391) | 0.007 | 1.103 (0.651 – 1.868) | 0.717 |
| mRSS | 1.031 (1.020 – 1.043) | <0.001 | 1.031 (1.002 – 1.062) | 0.035 |
| Esophageal symptoms | 1.046 (0.852 – 1.283) | 0.668 |  |  |
| Inflammatory Arthrits | 2.244 (1.696 – 2.696) | <0.001 | 3.034 (1.551 – 5.936) | 0.001 |
| Myositis | 1.395 (1.060 – 1.837) | 0.018 | 1.055 (0.479 – 2.321) | 0.894 |
| ACA | 0.846 (0.561 – 1.275) | 0.846 |  |  |
| ATA | 1.053 (0.830 – 1.336) | 0.670 |  |  |
| ARA | 1.190 (0.742 – 1.908) | 0.470 |  |  |
| CRP-elevation | 1.581 (1.076 – 2.323) | 0.020 | 1.237 (0.759 – 2.014) | 0.393 |
